# RGD-modified oncolytic adenovirus-harboring shPKM2 exhibits a potent cytotoxic effect in pancreatic cancer via autophagy inhibition and apoptosis promotion

**DOI:** 10.1038/cddis.2017.230

**Published:** 2017-06-01

**Authors:** Yanni Xu, Liang Chu, Sujing Yuan, Yuanqin Yang, Yu Yang, Bin Xu, Kangjian Zhang, Xin-Yuan Liu, Ruwei Wang, Ling Fang, Zhinan Chen, Zongsuo Liang

**Affiliations:** 1 College of Life Sciences, Northwest Agriculture and Forestry University, Yangling 712100, PR China; 2 Hepatic Surgery Center, Tongji Hospital, Tongji Medical College, Huazhong University of Science and Technology, Wuhan 430030, PR China; 3 State Key Laboratory of Cell Biology, CAS Center for Excellence in Molecular Cell Science, Shanghai Institute of Biochemistry and Cell Biology, Chinese Academy of Science, University of Chinese Academy of Science, Shanghai 200031, PR China; 4 Xinyuan Institute of Medicine and Biotechnology, Zhejiang Sci-Tech University, Hangzhou 310018, PR China; 5 Department of General Surgery, Shanghai 10th People’s Hospital, Tongji University School of Medicine, Shanghai 200072, PR China; 6 Zhejiang Conba Pharmaceutical Co., Ltd, Hangzhou 310018, PR China; 7 Department of Cell Biology, State Key Laboratory of Cancer Biology, Cell Engineering Research Center, Fourth Military Medical University, Xi’an 710032, PR China

## Abstract

The M2 isoform of pyruvate kinase (PKM2) is a key driver of glycolysis in cancer cells and has critical ‘non-metabolic’ functions in some cancers; however, the role of PKM2 in pancreatic cancer remains unclear. The aim of the current study was to elucidate the role of PKM2 in pancreatic cancer progression and the potential of PKM2 as a therapeutic target. In this study, we observed that PKM2 is highly expressed in patients with pancreatic cancer and is correlated to survival. Elevated PKM2 expression promoted cell proliferation, migration and tumor formation. The inhibition of cell growth by silencing PKM2 is caused by impairment of the autophagy process. To test the potential effects of downregulating PKM2 as a clinical therapy, we constructed an RGD-modified oncolytic adenovirus containing shPKM2 (O^Ad^.R.shPKM2) to knock down PKM2 in pancreatic cancer cells. Cells transduced with O^Ad^.R.shPKM2 exhibited decreased cell viability, and, in a PANC-1 xenograft model, intratumoral injection of O^Ad^.R.shPKM2 resulted in reduced tumor growth. Furthermore, O^Ad^.R.shPKM2 induced apoptosis and impaired autophagy in PANC-1 cells. Our results suggested that targeting PKM2 with an oncolytic adenovirus produced a strong antitumor effect, and that this strategy could broaden the therapeutic options for treating pancreatic cancer.

Pancreatic cancer is projected to become the second most-common cause of cancer-related death by 2030.^1^ It is nearly undetectable during the early stages, and advanced-stage disease is unresectable and lacks an effective treatment. Despite half a century of research and therapeutic development, the 5-year survival rate is less than 7%, and the median survival rate remains at 6 months.^2^ Therefore, it is critical to identify novel therapeutic targets and develop potential therapeutic strategies for pancreatic cancer.

Altered cellular metabolism is a hallmark of cancers.^3^ Unlike normal cells, cancer cells can shift their glucose metabolism towards glycolysis, even in an oxygenated environment. This phenomenon is characterized by increased glucose consumption and an elevated rate of lactate production and is known as aerobic glycolysis, or the ‘Warburg effect’.^4^ Pyruvate kinase is a key enzyme in glycolysis that regulates the final rate-limiting step of catalysing the transfer of a phosphate from phosphoenolpyruvate to adenosine diphosphate to produce pyruvate and energy (ATP). The pyruvate kinase gene comprises four isoenzymes encoded by two distinct genes in mammals, PKM and PKLR. The two splice variants of PKM pre-mRNA produce pyruvate kinase M1 (PKM1) and M2 (PKM2), which include exons 9 or 10, respectively.^5^ An increasing number of studies have shown that PKM2 but not PKM1 is crucial for tumorigenic phenotype maintenance, cell cycle progression and tumor growth.^6, 7^ Recently, PKM2 was identified as a protein kinase and transcription factor coactivator in regulating brain tumorigenesis and colon cancer cell migration, respectively, which are divergent from its canonical role as a pyruvate kinase.^8, 9^ Modulating PKM2 in tumor angiogenesis was recently reported to be regulated by miR-148a and miR-152 expression.^10^ PKM2 prevents apoptosis in hepatocellular carcinoma (HCC), and knockdown of PKM2 inhibited cell proliferation and induced apoptosis in HCC.^11^ The outcome of patients with either HCC or pancreatic cancer is inversely correlated with PKM2 expression.^11, 12, 13^ Because of its multiple roles in tumorigenesis, PKM2 should be investigated as a target for pancreatic cancer therapy.

Replication-selective oncolytic adenovirus carrying either therapeutic genes or shRNA has been shown to exert promising antitumor effects on different types of cancers.^14, 15^ For improved implementation of this vector as a cancer therapy, some modifications have been made. Replacing the original E1A promoter with a tumor-specific promoter can transcriptionally control viral replication to some extent.^16^ The vital gene for late viral RNA export is E1B 55K, and viruses with an E1B 55K deletion are incapable of replication in normal cells; however, tumor cells can efficiently export late viral RNA in the absence of E1B 55K.^17^ As a binding partner of adenovirus type 5, the coxsackie and adenovirus receptor (CAR), when expressed on tumor cells, restricts the infection efficiency of adenovirus type 5. We previously inserted an Arg-Gly-Asp (RGD) motif into the HI loop of the adenovirus knob, which significantly elevated the infection efficiency of the adenovirus.^18^

In the present study, we observed that PKM2 is overexpressed in pancreatic cancer samples and is correlated with patient survival. We showed that PKM2 knockdown inhibited cell proliferation, migration and tumor formation, and that PKM2 supressed autophagy in pancreatic cancer. We constructed an oncolytic adenovirus that expressed an shRNA targeting PKM2 (O^Ad^.R.shPKM2). Cells transduced with O^Ad^.R.shPKM2 exhibited increased apoptosis induction and reduced autophagy. This study indicated that PKM2 could be an effective therapeutic target for pancreatic cancer.

## Results

### PKM2 is highly expressed in pancreatic cancer samples and predicts poor survival

**Figure 1:**
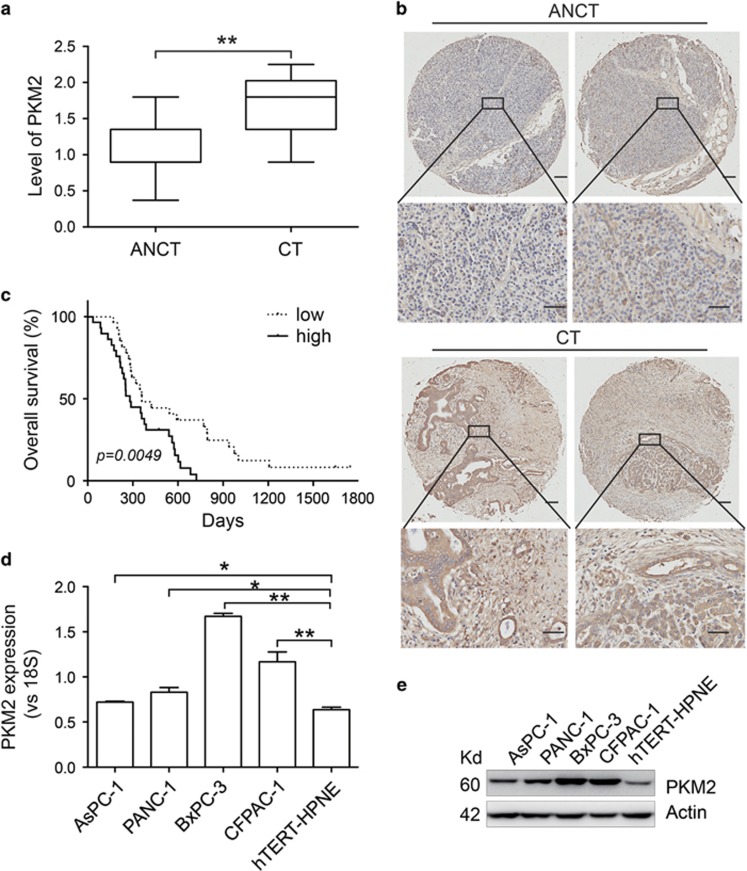
PKM2 is highly expressed in pancreatic cancer samples and predicts poor survival. (**a**) PKM2 expression was analyzed in pancreatic cancer tissue (CT, *n*=198) and adjacent noncancerous tissue specimens (ANCT, *n*=84) using IHC. (**b**) Representative IHC images. The scale bars represent 50 *μ*m. (**c**) Kaplan–Meier analysis to identify a correlation between PKM2 expression and overall survival of 60 pancreatic cancer patients. (**d** and **e**) PKM2 expression in the pancreatic cancer cell lines AsPC-1, PANC-1, BxPC-3 and CFPAC-1 as well as the normal pancreatic duct cell hTERT-HPNE was measured using qRT-PCR (**d**) and western blotting (**e**). The qRT-PCR data in (**d**) were normalized to 18S RNA levels. All experiments were repeated three times, and the bars represent the mean±S.D. (*n*=3); *P<0.05, **P<0.01

To determine whether PKM2 expression has clinical implications in human pancreatic cancer, we detected PKM2 expression in a tissue microarray containing 282 specimens (198 pancreatic cancer tissues and 84 adjacent noncancerous tissues) using immunohistochemistry (IHC). PKM2 expression in the pancreatic cancer tissues was significantly upregulated compared with that in the adjacent noncancerous tissues (Figures 1a and b). We further assessed the correlation between PKM2 expression and the clinical characteristics of pancreatic cancer patients (Table 1), and found that PKM2 expression was not related to gender or tumor stages but was significantly associated with age (*P*=0.0020) and lymph node metastasis (*P*=0.0436). High levels of PKM2 expression were correlated with worse overall survival in these pancreatic cancer patients (Figure 1c). In addition, PKM2 expression was higher in several pancreatic cancer cell lines (AsPC-1, PANC-1, BxPC-3 and CFPAC-1) than in the normal pancreatic duct cell line hTERT-HPNE (Figures 1d and e).

### PKM2 enhances pancreatic cancer cell proliferation and migration as well as tumor formation

**Figure 2:**
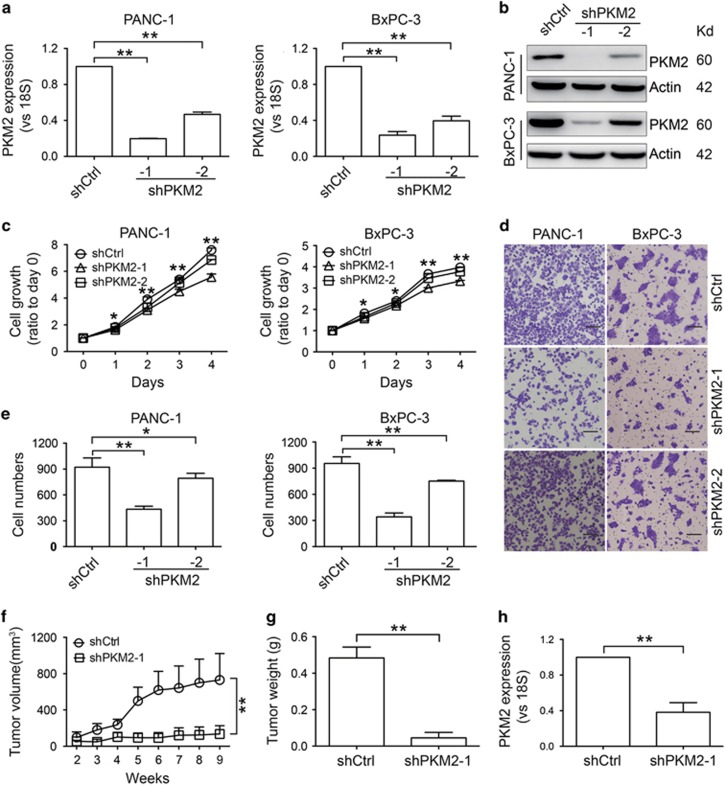
PKM2 enhances the proliferation, migration and tumor formation of pancreatic cancer cells. (**a** and **b**) qRT-PCR (**a**) and western blotting (**b**) showed reduced levels of PKM2 in PANC-1 and BxPC-3 cells expressing shPKM2. (**c**) PKM2 knockdown inhibited cell proliferation as measured using CCK-8 assays. (**d**) Transwell assays showed that PKM2 knockdown decreased PANC-1 and BxPC-3 cell migration. Scale bars, 200 *μ*m. (**e**) The number of migratory cells was counted. (**f**) The tumor growth curve showed that PANC-1 cells with PKM2 knockdown formed larger xenografts than control PANC-1 cells. (**g**) The mass of the tumors in each group was measured. (**h**) qRT-PCR detection of PKM2 expression in tumors. The qRT-PCR data in **a** and **h** were normalized to 18S levels and are shown as the fold change relative to PANC-1 cells transduced with shCtrl. All the experiments were repeated three times, and the bars represent the mean±S.D. (*n*=3 in **a**, **c**–**e**; *n*=4 in **f**–**h**). *P<0.05, **P<0.01

We constructed PKM2 knockdown cells by transducing PANC-1 and BxPC-3 cells with lentivirus containing PKM2-targeted shRNA. The levels of PKM2, but not PKM1, were significantly decreased in stably transduced cells, and shPKM2-1 exhibited better knockdown efficiency (Figures 2a, b, Supplementary Figures S1a and b). Moreover, knockdown of PKM2 decreased cyclin D1 expression, which is a downstream target of PKM2 (Supplementary Figure S1c).^8^ PKM2 knockdown significantly inhibited cell viability as measured by the CCK-8 and cell-counting assays (Figure 2c, Supplementary Figures S1d and e). Cell migration in PANC-1 and BxPC-3 cells with PKM2 knockdown was suppressed (Figures 2d and e). We then sought to test the ability of tumor formation in nude mice using PANC-1 shPKM2-1 and shCtrl cells, respectively. Silencing PKM2 significantly inhibited tumor growth (Figure 2f). The tumor mass and the PKM2 expression levels in tumors derived from cells expressing shPKM2-1 were significantly lower than those in the shCtrl group (Figures 2g and h). In addition, we found that all the mice bearing PANC-1 shCtrl tumors (4/4) but none of the mice bearing PANC-1 shPKM2-1 tumors (0/4) had liver metastases (Supplementary Figures S1f and g). The levels of aspartate aminotransferase (AST) and alanine aminotransferase (ALT) in the PANC-1 shPKM2-1 tumors were outside the normal range observed in the control group (Supplementary Figure S1h).

### Lentivirus-driven silencing of PKM2 impaired pancreatic cancer growth by inhibiting autophagy rather than inducing apoptosis

**Figure 3:**
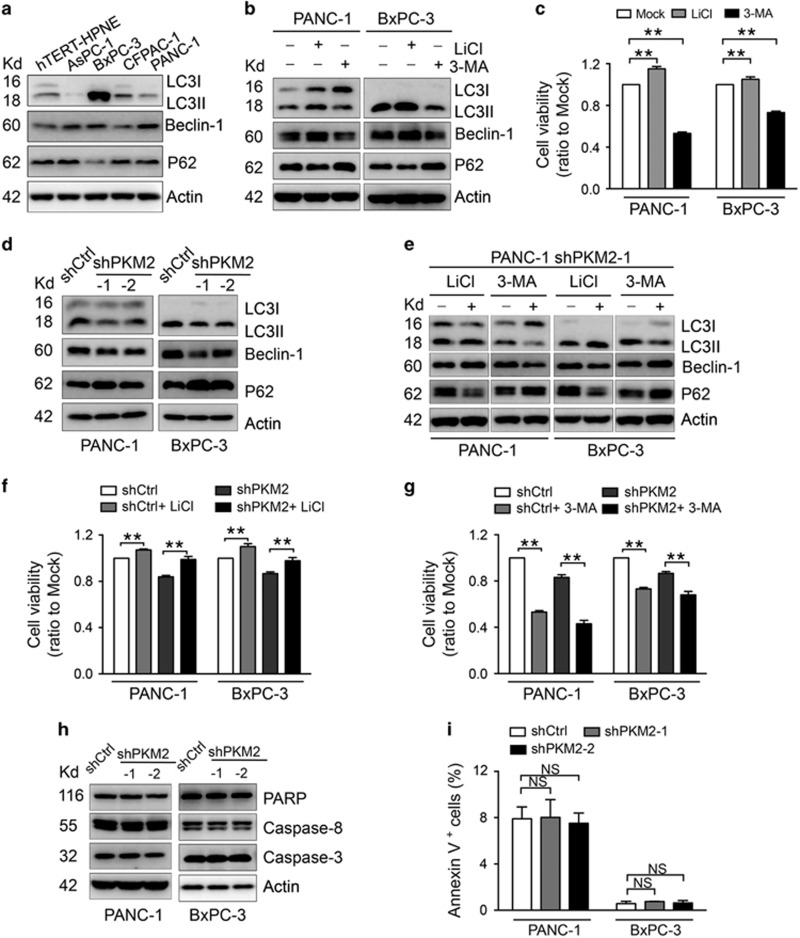
PKM2 silencing impairs pancreatic cancer growth by inhibiting autophagy rather than by inducing apoptosis. (**a**) Western blot detection of the LC3-I/II ratio, Beclin-1 and p62 in pancreatic cancer cells and normal hTERT-HPNE cells. (**b**) Western blot analysis of the LC3-I/II ratio, Beclin-1 and p62 expression in cells treated with either LiCl (1 mM) or 3-MA (5 mM) for 2 days. (**c**) The viability of cells treated with either LiCl (1 mM) or 3-MA (5 mM) for 2 days as measured by the CCK-8 assay. (**d**) Autophagy marker proteins were detected in PANC-1 and BxPC-3 cells stably transduced with shPKM2. (**e**) Autophagy marker proteins were detected in PANC-1 and BxPC-3 cells stably transduced with shPKM2 and treated with either LiCl (1 mM) or 3-MA (5 mM) for 2 days. (**f** and **g**) The viability of cells with PKM2 knockdown after treatment with either LiCl (1 mM; **f**) or 3-MA (5 mM; **g**) for 2 days. (**h**) Western blot detection of caspase-8, caspase-3 and PARP in PANC-1 and BxPC-3 cells stably transduced with shPKM2. (**i**) Apoptosis was evaluated in cells with stable knockdown of PKM2 using annexin V/PI staining. NS, no significance. Actin was used as a loading control in **a**, **b**, **d**, **e** and **h**). The cell viability in **c**, **f** and **g** was shown as the fold change relative to untreated mock cells. All experiments were repeated three times, and the bars represent the mean±S.D. (*n*=3). *P<0.05, **P<0.01

It has been reported that autophagy facilitates the acquisition of additional nutrition for pancreatic cancer cells to increase survival.^19, 20, 21, 22^ We detected the basal autophagy levels and observed a high LC3-II/LC3-I ratio as well as elevated Beclin-1 and lower SQSTM1/P62 levels, indicating that basal autophagy levels in pancreatic cancer cells are higher than those in normal cells (Figure 3a).^23, 24, 25^ The autophagy inhibitor 3-methyladenine (3-MA) remarkably declined PANC-1 and BxPC-3 cell proliferation. Conversely, the autophagy activator lithium chloride (LiCl) can accelerate cell proliferation (Figures 3b and c). Knockdown of PKM2 in PANC-1 and BxPC-3 cells caused a significant decrease in the LC3-II/LC3-I ratio and Beclin-1 expression in conjunction with P62 accumulation (Figure 3d). The number of LC3 puncta was reduced in cells with silenced PKM2 (Supplementary Figures S2a–c). A small number of autophagic structures was found in PKM2-knockdown cells (Supplementary Figure S2d), and the mRNA and protein expression levels of the autophagy-related transcription factors HIF-1*α* and FoxO3a were both reduced in PKM2-knockdown cells (Supplementary Figures S2e–g). LiCl reversed the inhibition of cell proliferation caused by PKM2 knockdown (Figures 3e and f); cell growth was further inhibited by 3-MA in the same cells (Figures 3e and g). These results suggested that PKM2 knockdown inhibited cell growth by suppressing autophagy. We next investigated whether PKM2 knockdown could induce cell apoptosis. Compared to cells with native PKM2 levels, cells with lentivirus-driven PKM2 silencing showed no difference in apoptotic induction (Figures 3h, i and Supplementary Figure S2h), indicating that the inhibition of pancreatic cancer cell growth by lentivirus-mediated silencing of PKM2 does not occur through apoptosis.

### An RGD-modified oncolytic adenovirus carrying a PKM2-targeted shRNA exerts enhanced cytotoxic effects on pancreatic cancer cells

**Figure 4:**
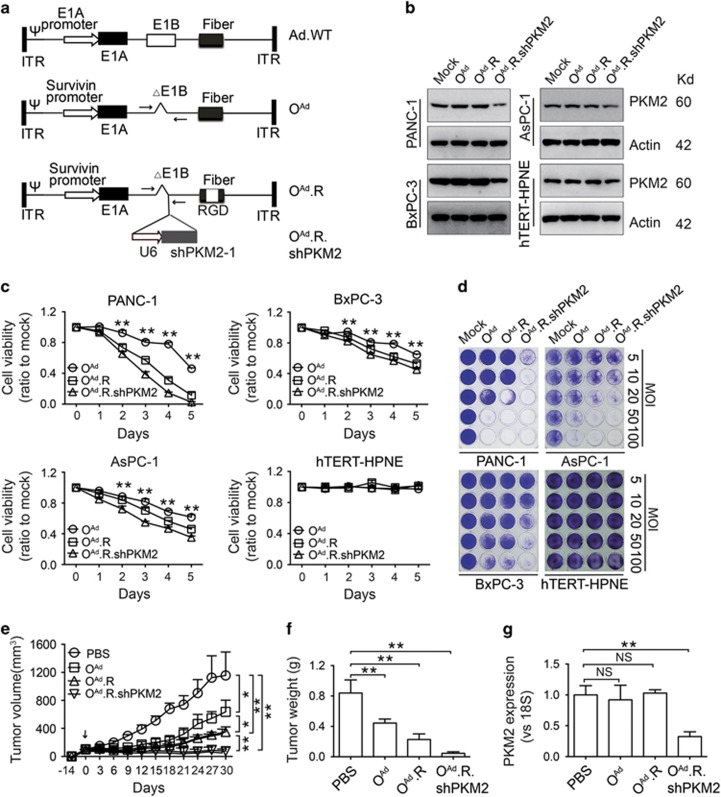
RGD-modified oncolytic adenovirus carrying shPKM2 exerts an enhanced cytotoxic effect in pancreatic cancer cells. (**a**) Schematic construction of the oncolytic adenoviruses. Arrows indicate the primers used to detect wild-type adenovirus contamination. (**b**) PKM2 expression was detected in cells infected with the indicated oncolytic adenovirus for 2 days at an MOI of 10. (**c**) The viability of cells infected with the indicated oncolytic adenoviruses at an MOI of 10 was assessed using CCK-8 assays. (**d**) The viability of cells infected with O^Ad^.R.shPKM2 at the indicated MOIs for 5 days was measured using crystal violet staining. (**e**) The growth curve of PANC-1 xenograft tumors after intratumoral injection of the indicated adenoviruses. Arrows represent the action of the intratumoral injection. (**f**) Tumors were excised and weighed 30 days after initial injection. (**g**) qRT-PCR detection of PKM2 expression in tumors. The data were normalized to 18S levels and are shown as the fold change relative to PANC-1 shCtrl cells. All experiments were repeated three times, and the bars represent the mean±S.D. (*n*=3 in **c**, *n*=6 in **e**–**g**), *P<0.05, **P<0.01. NS, no significance

Because pancreatic cancer cells express low levels of CAR (Supplementary Figures S3a and b), we constructed an RGD-modified oncolytic adenovirus carrying PKM2-targted shRNA (O^Ad^.R.shPKM2) to investigate whether PKM2 could be a therapeutic target for pancreatic cancer (Figure 4a and Supplementary Figure S3c). The RGD-modified virus contains a short sequence encoding the CDCRGDCFC (RGD-4C) peptide in the HI loop of the fiber-coding region. The shPKM2 sequence was inserted into the E1B deletion region. PCR amplification and sequencing of the resulting products confirmed the insertion of RGD (Supplementary Figure S3d) without any contamination of wild-type adenovirus (Supplementary Figure S3e). We first detected the infectiousness of the RGD-modified oncolytic adenovirus. As expected, there were significantly more EGFP-positive cells among PANC-1 cells infected with O^Ad^.R.EGFP than among cells treated with O^Ad^.EGFP (Supplementary Figures S3f and g), indicating the superior infectious ability of the RGD-modified oncolytic adenovirus. In addition, O^Ad^.R.shPKM2 downregulated PKM2 expression in pancreatic cancer cells but not in hTERT-HPNE cells (Figure 4b). O^Ad^.R inhibited cell proliferation, an effect that was further enhanced by O^Ad^.R.shPKM2 (Figures 4c and d).

We then subcutaneously injected PANC-1 cells into nude mice. When the tumors grew to ~100 mm^3^, O^Ad^, O^Ad^.R, O^Ad^.R.shPKM2 or the corresponding volume of PBS were intratumorally injected. At 30 days after treatment injection, the mice were killed, and the tumor mass was measured. The results showed that O^Ad^.R.shPKM2 suppressed tumor growth and that the tumor mass was smaller in the O^Ad^.R.shPKM2 treatment group (Figures 4e–g and Supplementary Figure S3h), indicating that O^Ad^.R.shPKM2 significantly inhibited tumor growth. No significant changes in the AST and ALT levels were observed after virus treatment (Supplementary Figure S3i).

### O^Ad^.R.shPKM2 promotes cell apoptosis and inhibits cell autophagy

**Figure 5:**
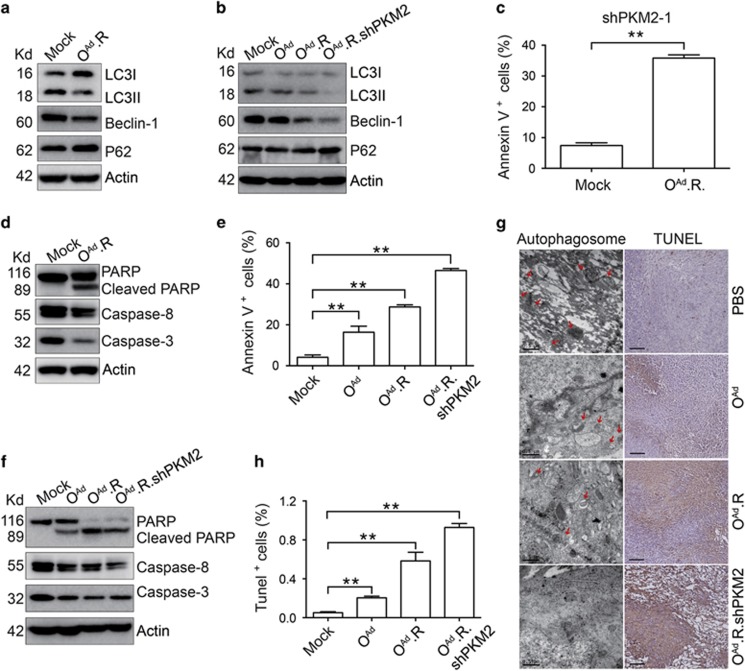
O^Ad^.R.shPKM2 promotes apoptosis and inhibits autophagy in PANC-1 cells. (**a**) Western blot detection of the protein expression levels of the LC3-I/II ratio, Beclin-1 and p62 in PANC-1-shPKM2 cells treated with O^Ad^.R at an MOI of 10 for 2 days. (**b**) Western blot detection of the LC3-I/II ratio, Beclin-1 and p62 in oncolytic adenovirus-infected PANC-1 cells at an MOI of 10 for 2 days. (**c**) Apoptosis was evaluated in PANC-1-shPKM2 cells treated with O^Ad^.R at an MOI of 10 for 2 days using annexin V/PI staining. (**d**) Western blot detection of caspase-8, caspase-3 and PARP in PANC-1-shPKM2 cells treated with O^Ad^.R at an MOI of 10 for 2 days. (**e**) Apoptosis was evaluated in oncolytic adenovirus-infected PANC-1 cells using annexin V/PI staining. (**f**) Western blot detection of caspase-8, caspase-3 and PARP in oncolytic adenovirus-infected PANC-1 cells. Cells were infected with the indicated oncolytic adenoviruses at an MOI of 10 for 2 days. (**g**) Representative images of TUNEL staining (scale bar, 200 *μ*m; right panel) and electron microscopy (scale bar, 500 nm; left panel) of the tumor sections from each group. The red arrows indicate the autophagosome. (**h**) Quantification of the TUNEL staining results observed in **g**. Actin was used as a loading control in **a**, **b**, **d** and **f**. All experiments were repeated three times, and the bars represent the mean±S.D. (*n*=3). **P<0.01

The O^Ad^.R virus inhibited autophagy in PANC-1 cells infected with lentivirus containing shPKM2 (Figure 5a), and this autophagy was distinctly inhibited by O^Ad^.R.shPKM2 treatment (Figure 5b and Supplementary Figures S4a–c). Interestingly, we found that O^Ad^.R could induce apoptosis in shPKM2-transduced PANC-1 cells (Figures 5c, d and Supplementary Figure S4d). The number of annexin V-positive cells was remarkably elevated after O^Ad^.R.shPKM2 treatment (Figure 5e and Supplementary Figure S4e); additionally, the protein levels of procaspase-8 and procaspase-3 were reduced, and the cleaved form of PARP protein was increased (Figure 5f), indicating that O^Ad^.R.shPKM2 could induce cell apoptosis.

We next examined whether O^Ad^.R.shPKM2 could inhibit autophagy and induce apoptosis in PANC-1 xenograft tumors by using transmission electron microscopy and TUNEL staining, respectively. The number of autophagic structures was decreased in tumors treated with oncolytic adenovirus, and a few autophagic structures were observed in tumors treated with O^Ad^.R.shPKM2 (Figure 5g). The percentage of TUNEL-positive cells in the O^Ad^.R.shPKM2 group was significantly higher than that in the O^Ad^ and O^Ad^.R groups (Figures 5g and h).

## Discussion

Here, we showed that PKM2 is highly expressed in pancreatic cancers and is associated with poor patient survival. We also found that PKM2 modulated cell proliferation and migration as well as tumor formation of pancreatic cancer. A mild reduction in autophagy was observed in PANC-1-shPKM2 cells, indicating that impaired autophagy was mediated by PKM2 knockdown in cells with high autophagic flux. Targeting PKM2 using an RGD-modified oncolytic adenovirus (O^Ad^.R.shPKM2) displayed a potent cell cytotoxic effect. O^Ad^.R.shPKM2 induced apoptosis and inhibited autophagy both *in vitro* and *in vivo*.

We detected the PKM1 and PKM2 expression levels in pancreatic cancer cell lines and in patient samples (Figure 1 and Supplementary Figure S5), and found that PKM2 but not PKM1 was related to patient survival, which was in agreement with the results of other studies (Figure 1c and Supplementary Figure S5c).^12^ Our results are also consistent with previous studies reporting that PKM2 levels are increased in other cancer types such as HCC, pulmonary adenocarcinoma, tongue cancer and glioma.^12, 26, 27, 28, 29^ Older pancreatic cancer patients expressed lower levels of PKM2 (Table 1). PKM expression is tissue-specific and is controlled by development, diet and hormones,^30, 31^ which may explain why PKM2 expression decreases with age. Importantly, our data indicated that all patients with lymph node metastasis displayed elevated PKM2 expression. Pancreatic cancer cells lacking PKM2 knockdown also showed enhanced migratory capabilities (Figures 2d and e). Mice bearing tumors with low levels of PKM2 expression had no liver metastases (Supplementary Figures S1f and g). The oncolytic adenovirus-treated mice were killed less than 2 months, and no metastases were found. We speculated that this timeframe was too short to form measurable metastases. PKM2-mediated regulation of migration in other cancers has also been reported. PKM2 overexpression was shown to promote colon cancer cell migration and cell adhesion by regulating STAT3-associated signaling.^9^ In addition, downregulation of PKM2 by miR-let-7a inhibited migration in gastric cancer.^32^ However, elucidating the underlying mechanism of PKM2 in regulating pancreatic cancer metastasis requires further investigation.

Our results showed that PKM2 could promote pancreatic cancer progression (Figures 2c–g), which was similar with previous studies of other cancer types such as non-small cell lung carcinoma, myeloma and gastric cancer.^7, 32, 33^ Our data also revealed that PKM2 knockdown inhibited autophagy in pancreatic cancer cells *in vitro* and *in vivo* (Figures 3d, 5g and Supplementary Figures S2a–d). The expression levels of the autophagy-related factors HIF-1*α* and FoxO3a were decreased in PKM2-knockdown cells (Supplementary Figures S2e–g). PKM2 is a target of HIF-1*α*, and PKM2 expression can influence the transcription and transcriptional activity of HIF-1*α* as a form of feedback.^34^ HIF-1*α* activates the transcription of the gene encoding BNIP3, a BH3 domain protein that induces selective mitochondrial autophagy by binding to Bcl-2 to release Beclin-1.^35^ It was reported that downregulation of HIF-1*α* by siRNA could decrease autophagy by reducing the levels of free Beclin-1.^36^ FoxO3a localizes to the cell nucleus and activates the expression of genes involved in autophagy pathways.^37^ Moreover, FoxO3a is able to induce the transcription of target genes involved in cell cycle arrest, cell death, cell metabolism and stress resistance.^38, 39^ In cells depleted of energy due to blocked p38*α* activity, FoxO3a was upregulated and autophagy was induced.^40^ In pancreatic cancer cells with higher basal levels of autophagy (Figure 3a), PKM2 knockdown reduced the expression of FoxO3a and impaired autophagy (Supplementary Figures S2f–g). However, how FoxO3a is regulated in pancreatic cancer cells is still unclear, and the mechanism by which PKM2 regulates autophagy still requires validation with additional experiments.

Oncolytic adenovirus is an attractive vector that can selectively replicate in tumor cells and can constantly express either therapeutic proteins or shRNA with virus replication. Adenovirus modifications include (1) an RGD motif containing a peptide sequence to improve viral insertion into the target cell; (2) a survivin promoter driving E1A gene expression; and (3) loss of the regulation of viral E1B 55K RNA export and the apoptosis-restraining domain of E1B 19K.^41, 42, 43^ Owing to the low CAR expression levels in pancreatic cancer cells (Supplementary Figures S3a and b), the adenovirus infection efficiency was poor. The RGD modification significantly increased the ability of adenovirus to enter cells with low levels of CAR expression (Supplementary Figures S3f and g). Cells transduced with a survivin promoter-driven oncolytic adenovirus displayed increased clinical safety. Utilizing a pancreatic cancer-specific promoter such as the Mucin1 Promoter (MUC1) to control E1A expression also enhanced the replication selectivity in pancreatic cancer.^44^ Systemic administration of oncolytic adenoviruses has favorable toxicity profiles in cancer patients. DNX-2401, which has received fast-track FDA approval, is an orphan drug for the treatment of malignant glioma.^45^ In addition, the development of other oncolytic viruses has recently made great strides toward availability for treating cancer patients. Talimogene laherparepvec (T-VEC; IMLYGIC, Amgen, Thousand Oaks, CA, USA) is an oncolytic herpes simplex virus containing granulocyte macrophage colony-stimulating factor (GM-CSF). This virus was tested in patients with unresected melanoma (stages IIIB to IV) in a randomized open-label phase III trial, and was compared to the effects of GM-CSF alone. T-VEC in combination with ipilimumab was administered to previously untreated patients with unresectable stage IIIB–IV melanoma. An intratumoral reovirus infusion for the treatment of recurrent malignant gliomas is currently in a phase I clinical trial.^46, 47, 48^

Calabretta *et al.*^13^ observed that knockdown of PKM2 enhanced the sensitivity of pancreatic cancer cells to chemotherapy-induced apoptosis. In this study, lentiviral-mediated knockdown of PKM2 had no effect on apoptosis compared to the control cells (Figures 3h and i). We also found that O^Ad^.R remarkably enhanced apoptosis in PANC-1 cells infected with lentivirus containing shPKM2 (Figures 5c, d and Supplementary Figure S4d). It has been reported that oncolytic adenovirus can cause apoptosis in various cancer cells.^16, 18, 49^ Therefore, we speculated that oncolytic adenovirus might trigger apoptosis in cells with adenoviral vector-mediated PKM2 knockdown. There exists complex crosstalk between autophagy and apoptosis because they share common stimuli and signaling pathways.^50^ Induction of apoptosis is associated with caspase-mediated cleavage of Beclin-1 and PI3K,^51, 52, 53, 54^ an event that impairs the autophagic function of Beclin-1. In addition, recent studies have shown that caspase-8 activation can be degraded by autophagy,^24^ which further suggests the existence of a feedback mechanism that regulates both autophagy and apoptosis. On the basis of this notion, the apoptosis induced by adenoviral vectors may facilitate the ongoing feedback between autophagy and apoptosis. Hence, targeting the crosstalk between autophagy and apoptosis is a promising new approach for pancreatic cancer therapy.

In all, our data showed that PKM2 is critical in pancreatic cancer and is related to cell proliferation, migration and metastasis. PKM2 regulated cell growth by inhibiting autophagy. Expression of O^Ad^.R.shPKM2 in cells exhibited robust effects *in vitro* and *in vivo* by inducing apoptosis and inhibiting autophagy, suggesting a potential antitumor therapy for pancreatic cancer.

## Materials and methods

### Patients and tissue microarray

Tissue microarrays consisting of 198 pancreatic cancer and 84 adjacent normal specimens were provided by Bin Xu. The pathological information was retrieved from the Pathology Department of Shanghai 10th People’s Hospital. The tumors were categorized as clinical stages I, II, III and IV based on the seventh edition of the American Joint Committee on Cancer criteria. All the human samples were collected after patients provided informed consent, and the approval for specimen usage was obtained from the ethics committee at the 10th People’s Hospital. Studies involving these samples were approved and handled in accordance with the Institutional Review Board of the Institute of Biochemistry and Cell Biochemistry, Shanghai Institutes for Biological Sciences, Chinese Academy of Sciences.

### Immunohistochemistry

The sections of tissue microarrays were subjected to IHC assay according to the established protocols. The stained tissue microarrays were analyzed by a pathologist blinded to the patient status. The levels of PKM2 and PKM1 expression were defined as values ranging from 0 to 3 according to the intensity, and quantification of PKM2 (Signalway Antibody, Washington, MD, USA) and PKM1 (Proteintech, Chicago, IL, USA) expression was calculated as (labeling intensity × percentage of positive cells).

### Cell culture

The human pancreatic cancer cell lines PANC-1, BxPC-3, AsPC-1 and CFPAC-1, the human HCC cell line QGY-7701 and HEK-293T cells were purchased from the Cell Bank of the Type Culture Collection of the Chinese Academy of Sciences (Shanghai, China). The HEK-293 cell line was obtained from Microbix Biosystems Inc. (Toronto, ON, Canada). The human pancreatic duct epithelial-like cell line hTERT-HPNE was purchased from the American Type Culture Collection. All cells were cultured according to the manufacturer’s instructions.

### Establishing cells with stable PKM2 knockdown

shRNAs against PKM2 (shPKM2-1: 5′-CATCTACCACTTGCAATTA-3′, shPKM2-2: 5′-CCATAATCGTCCTCACCAA-3′) as well as a scramble control shRNA (5′-CTACCGTTGTTATAGGTG-3′) were synthesized and cloned into the pLVX vector according to the standard protocols. Lentivirus was produced in 293T cells by transfecting pLVX-shPKM2 and the corresponding lentiviral helper plasmids, and target cells were infected with the lentiviral vectors and selected using a specific concentration of puromycin.

### Quantitative RT-PCR

Total RNA was isolated using TRIzol reagent (CWBIO, Beijing, China), and single-strand cDNA was synthesized using a ReverTra Ace qPCR RT kit (Toyobo, Osaka, Japan). RNA expression was analyzed using SuperReal Premix Plus (TIANGEN, Beijing, China) according to the manufacturer’s protocols. The sequences of all the primers are shown in Supplementary Table S1. Each assay was performed in triplicate.

### Western blotting

Cell lysates were collected in cell lysis buffer (Beyotime, Shanghai, China) containing a complete protease inhibitor tablet (Roche, Basel, Switzerland) and 1 mM phenylmethanesulfonylfluoride. The protein levels were quantified with a Lowry kit (Bio-Rad, Berkeley, CA, USA) according to the manufacturer’s instructions. PVDF membranes (Millipore, Billerica, MA, USA) were incubated with the following antibodies: anti-PKM2 (SAB, Washington, MD, USA); anti-PKM1 (Proteintech); anti-HIF-1*α* (Cell Signaling Technology (CST), MA, USA); anti-FoxO3a (Abcam, Cambridge, UK); anti-procaspase-8, anti-procaspase-3, anti-PARP and anti-Beclin-1 (Santa Cruz Biotechnology, Santa Cruz, CA, USA); anti-cyclin D1 (CST); anti-actin (CWBIO, Beijing, China); and anti-LC3 and anti-p62 (Sigma, St. Louis, MO, USA). All HRP-conjugated secondary antibodies were purchased from Santa Cruz Biotechnology. Protein detection was performed using either Pierce ECL (CWBIO, Beijing, China) or Pierce SuperSignal Pico (Thermo Fisher Scientific, USA) reagents.

### Proliferation assays

Cell proliferation was measured using a CCK-8 kit (Dojindo, Kumamoto, Japan). Cells were incubated with 10 *μ*l of CCK-8 at 37 °C for 1.5 h, and the absorbance at wavelengths of 450 and 630 nm were applied using a BioTek Eon Microplate Reader (BioTek, Washington, CA, USA).

### High-content cell number counting

Cells were fixed with 4% paraformaldehyde and labeled with 10 *μ*M Hoechst (Thermo Fisher Scientific) to stain DNA. Fluorescent images were analyzed using an Operetta high-content imaging system (PerkinElmer, Waltham, MA, USA) at × 20 magnification. Different parameters of the cytoskeletal changes were measured using Harmony High Content Imaging and Analysis Software (PerkinElmer).

### Crystal violet nucleus staining

After the cells were exposed to 2% crystal violet in 20% methanol for 15 min, they were washed with distilled water and photographed.

### Migration assays

Cell migration assays were performed using transwell inserts (8 *μ*m, BD Biosciences, San Jose, CA, USA) placed in 24-well plates. A total of 8 × 10^4^ cells were plated in the upper chambers of the transwell inserts in 200 *μ*l of serum-free medium, whereas the medium in the bottom chamber contained 15% fetal bovine serum. Following a 20 h incubation at 37 °C, the inserts were stained with 0.5% crystal violet and gently washed, followed by imaging with an Olympus camera and counting using Image-Pro Plus 7.0 software (Media Cybernetics, Rockville, MD, USA).

### Immunofluorescence microscopy

Cells were fixed with 4% (w/v) paraformaldehyde, permeabilized using 0.1% (w/v) Triton X-100 and blocked with 1% bovine serum albumin. The cells were stained overnight with an anti-LC3 antibody (Sigma) and then incubated for 1 h at room temperature with a Cy3-conjugated secondary antibody (Abcam) diluted in PBS containing 1% FBS. Images were captured using a laser scanning confocal microscope.

### Adenovirus construction and identification

The expression cassette of shPKM2 was inserted into the deleted-E1B region of a shuttle vector (pShuttle-survivin-E1A-E1B). Different oncolytic adenoviral plasmids were generated using homologous recombination of the shuttle vector and adenoviral backbone plasmid in the *Escherichia coli* strain BJ5183. Viruses were packaged and amplified in HEK-293 cells followed by centrifugation in a graded CsCl solution for purification. The viral titer was measured using a QuickTiter Adenovirus Titer Immunoassay Kit (Cell Biolabs, San Diego, CA, USA). Viral genomes for identification were extracted using the Blood Genome Extract Kit (Generay, Shanghai, China) according to the manufacturer’s instructions. The existence of the RGD peptide-coding sequence and the E1B deletion and wild-type contaminants were shown using PCR and sequencing with the appropriate primers (Supplementary Table S1). PKM2 gene expression was examined using western blotting.

### Apoptosis detection

Cell apoptosis analysis was conducted using a fluorescein isothiocyanate-conjugated Annexin V (Annexin V-FITC)/Propidium Iodide Apoptosis Detection kit (BD Biosciences) according to the manufacturer’s instructions. Cells were analyzed using a BD LSRII flow cytometer, and all the experiments were repeated three times.

### Animal experiments

All animal experiments were performed according to protocols approved by the US Public Health Service Policy on Humane Care and Use of Laboratory Animals. Four-week-old female BALB/c nude mice were purchased from the Animal Core Facility (Shanghai, China).

For the tumorigenicity assay, 5 × 10^6^ PANC-1 shCtrl or shPKM2-1 cells were mixed with Matrigel (BD Biosciences) at a ratio of 2 : 1 and subcutaneously injected into the right and left rear back regions of mice, respectively. The tumor volume was measured every 7 days using a Vernier caliper and calculated using the following equation: (length × width^2^)/2.

To determine the *in vivo* antitumor effect of adenoviruses on PANC-1 xenografts, we premixed 5 × 10^6^ PANC-1 cells with Matrigel at a ratio of 1 : 2 and subcutaneously injected the mixture into the right rear back region of each mouse. When the tumors reached 80–120 mm^3^ in size, the mice were randomly divided into four groups (six mice per group) using R software (The University of Auckland, Auckland, New Zealand). Either oncolytic adenovirus (2.5 × 10^8^ PFU per mouse) or PBS was intratumorally injected every other day (four injections total), and the tumor volume was measured every 3 days. At the end of the experiment, the tumors were resected from the killed mice for histology (hematoxylin and eosin (H&E) staining) and immunofluorescence staining analyses. The serum levels of AST and ALT were also measured.

### Histological analysis and TUNEL staining

Formalin-fixed tissues were paraffin-embedded and sliced into 5-*μ*m sections stained using H&E. The TUNEL assay was performed using an *in situ* cell death detection kit (Roche) according to the manufacturer’s instructions. Images were captured using an Olympus BX51 microscope (Olympus Optical, Tokyo, Japan). The number of TUNEL-positive cells was counted in six random fields on each coverslip (two per condition), and the average number of TUNEL-positive cells per random field was determined for each condition tested and quantified using Image-Pro Plus 7.0 software.

### Transmission electron microscopy

Tumor tissues were fixed with 2.5% glutaraldehyde overnight, followed by 1% osmium tetroxide for 1.5 h. The tissue samples were then dehydrated using a graded series of ethanol, rinsed with acetone and permeabilized overnight with embedding buffer. Sections (70-nm thickness) were dual-stained with 2% uranyl acetate and lead citrate. Autophagosomes were observed using transmission electron microscopy (FEI, Hillsboro, OR, USA).

### Statistical analysis

Data are presented as the mean±S.D. Comparisons between two groups were performed using the unpaired Student’s *t*-test. The survival rate was calculated using Kaplan–Meier analysis. All tests were conducted using GraphPad Prism 6.0 software (GraphPad Software, LaJolla, CA, USA).

## Figures and Tables

**Table 1:** Correlation between PKM2 expression and the clinicopathological features of pancreatic cancer patients

**Variables**	**Total**		**Low PKM2**		**High PKM2**		***P*****-value**
	***N***	**%**	***N***	**%**	***N***	**%**	
*Tissue type*							**0.0013**
Adjacent	10	14.3	10	27.0	0	0.0	
Tumor	60	85.7	27	73.0	33	100.0	
							
*Age*							**0.0020**
<62	31	51.7	8	29.6	23	69.7	
>62	29	48.3	19	70.4	10	30.3	
							
*Gender*							0.8516
Male	37	61.7	17	63.0	20	60.6	
Female	23	38.3	10	37.0	13	39.4	
							
*Lymph node metastasis*							**0.0436**
Positive	5	11.6	0	0.0	5	20.0	
Negative	38	88.4	18	100.0	20	80.0	
							
*TNM stage*							0.4533
I	11	18.3	4	14.8	7	21.2	
II	43	71.6	20	74.1	23	69.7	
III	3	5.0	1	3.7	2	6.1	
IV	3	5.0	2	7.4	1	3.0	

*X*^2^-tests were used to analyze the correlation between PKM2 expression levels and clinical features. The median expression level was set as the cutoff value. Low levels of PKM2 expression in 27 patients were classified as values below the 50th percentile, whereas high levels of PKM2 expression in 33 patients were classified as values at or above the 50th percentile. Bold values had statistically significant differences.

## References

[bib1] Rahib L, Smith BD, Aizenberg R, Rosenzweig AB, Fleshman JM, Matrisian LM. Projecting cancer incidence and deaths to 2030: the unexpected burden of thyroid, liver, and pancreas cancers in the United States. Cancer Res 2014; 74: 2913–2921. 24840647 10.1158/0008-5472.CAN-14-0155

[bib2] Vogelzang NJ, Benowitz SI, Adams S, Aghajanian C, Chang SM, Dreyer ZE et al. Clinical cancer advances 2011: Annual Report on Progress Against Cancer from the American Society of Clinical Oncology. J Clin Oncol 2012; 30: 88–109. 22147736 10.1200/JCO.2011.40.1919

[bib3] Hanahan D, Weinberg RA. Hallmarks of cancer: the next generation. Cell 2011; 144: 646–674. 21376230 10.1016/j.cell.2011.02.013

[bib4] Vander Heiden MG, Cantley LC, Thompson CB. Understanding the Warburg effect: the metabolic requirements of cell proliferation. Science 2009; 324: 1029–1033. 19460998 10.1126/science.1160809 PMC2849637

[bib5] Macintyre AN, Rathmell JC. PKM2 and the tricky balance of growth and energy in cancer. Mol Cell 2011; 42: 713–714. 21700216 10.1016/j.molcel.2011.06.003 PMC3267323

[bib6] Gao X, Wang H, Yang JJ, Liu X, Liu ZR. Pyruvate kinase M2 regulates gene transcription by acting as a protein kinase. Mol Cell 2012; 45: 598–609. 22306293 10.1016/j.molcel.2012.01.001 PMC3299833

[bib7] Christofk HR, Vander Heiden MG, Harris MH, Ramanathan A, Gerszten RE, Wei R et al. The M2 splice isoform of pyruvate kinase is important for cancer metabolism and tumour growth. Nature 2008; 452: 230–233. 18337823 10.1038/nature06734

[bib8] Yang W, Xia Y, Hawke D, Li X, Liang J, Xing D et al. PKM2 phosphorylates histone H3 and promotes gene transcription and tumorigenesis. Cell 2012; 150: 685–696. 22901803 10.1016/j.cell.2012.07.018 PMC3431020

[bib9] Yang P, Li Z, Fu R, Wu H, Li Z. Pyruvate kinase M2 facilitates colon cancer cell migration via the modulation of STAT3 signalling. Cell Signal 2014; 26: 1853–1862. 24686087 10.1016/j.cellsig.2014.03.020

[bib10] Xu Q, Liu LZ, Yin Y, He J, Li Q, Qian X et al. Regulatory circuit of PKM2/NF-kappaB/miR-148a/152-modulated tumor angiogenesis and cancer progression. Oncogene 2015; 34: 5482–5493. 25703326 10.1038/onc.2015.6

[bib11] Hu W, Lu SX, Li M, Zhang C, Liu LL, Fu J et al. Pyruvate kinase M2 prevents apoptosis via modulating Bim stability and associates with poor outcome in hepatocellular carcinoma. Oncotarget 2015; 6: 6570–6583. 25788265 10.18632/oncotarget.3262 PMC4466635

[bib12] Mohammad GH, Olde Damink SW, Malago M, Dhar DK, Pereira SP. Pyruvate kinase M2 and lactate dehydrogenase A are overexpressed in pancreatic cancer and correlate with poor outcome. PLoS ONE 2016; 11: e0151635. 26989901 10.1371/journal.pone.0151635 PMC4798246

[bib13] Calabretta S, Bielli P, Passacantilli I, Pilozzi E, Fendrich V, Capurso G et al. Modulation of PKM alternative splicing by PTBP1 promotes gemcitabine resistance in pancreatic cancer cells. Oncogene 2016; 35: 2031–2039. 26234680 10.1038/onc.2015.270 PMC4650269

[bib14] Lee JS, Oh E, Yoo JY, Choi KS, Yoon MJ, Yun CO. Adenovirus expressing dual c-Met-specific shRNA exhibits potent antitumor effect through autophagic cell death accompanied by senescence-like phenotypes in glioblastoma cells. Oncotarget 2015; 6: 4051–4065. 10.18632/oncotarget.3018 PMC4414172 25726528

[bib15] Ma L, Liu J, Shen J, Liu L, Wu J, Li W et al. Expression of miR-122 mediated by adenoviral vector induces apoptosis and cell cycle arrest of cancer cells. Cancer Biol Ther 2010; 9: 554–561. 20150764 10.4161/cbt.9.7.11267

[bib16] Zhang KJ, Zhang J, Wu YM, Qian J, Liu XJ, Yan LC et al. Complete eradication of hepatomas using an oncolytic adenovirus containing AFP promoter controlling E1A and an E1B deletion to drive IL-24 expression. Cancer Gene Ther 2012; 19: 619–629. 22790965 10.1038/cgt.2012.40

[bib17] O’Shea CC, Johnson L, Bagus B, Choi S, Nicholas C, Shen A et al. Late viral RNA export, rather than p53 inactivation, determines ONYX-015 tumor selectivity. Cancer Cell 2004; 6: 611–623. 15607965 10.1016/j.ccr.2004.11.012

[bib18] Yang Y, Xu H, Shen J, Yang Y, Wu S, Xiao J et al. RGD-modifided oncolytic adenovirus exhibited potent cytotoxic effect on CAR-negative bladder cancer-initiating cells. Cell Death Dis 2015; 6: e1760. 25973680 10.1038/cddis.2015.128 PMC4669706

[bib19] White E, DiPaola RS. The double-edged sword of autophagy modulation in cancer. Clin Cancer Res 2009; 15: 5308–5316. 19706824 10.1158/1078-0432.CCR-07-5023 PMC2737083

[bib20] White E. Deconvoluting the context-dependent role for autophagy in cancer. Nat Rev Cancer 2012; 12: 401–410. 22534666 10.1038/nrc3262 PMC3664381

[bib21] White E, Mehnert JM, Chan CS. Autophagy, metabolism, and cancer. Clin Cancer Res 2015; 21: 5037–5046. 26567363 10.1158/1078-0432.CCR-15-0490 PMC4646728

[bib22] Perera RM. Transcriptional control of autophagy–lysosome function drives pancreatic cancer metabolism. Nature 2015; 524: 361–365. 26168401 10.1038/nature14587 PMC5086585

[bib23] Dey A, Mustafi SB, Saha S, Kumar Dhar Dwivedi S, Mukherjee P, Bhattacharya R. Inhibition of BMI1 induces autophagy-mediated necroptosis. Autophagy 2016; 12: 659–670. 27050456 10.1080/15548627.2016.1147670 PMC4836029

[bib24] Liu Y, Levine B. Autosis and autophagic cell death: the dark side of autophagy. Cell Death Differ 2015; 22: 367–376. 25257169 10.1038/cdd.2014.143 PMC4326571

[bib25] Komatsu M, Waguri S, Koike M, Sou YS, Ueno T, Hara T et al. Homeostatic levels of p62 control cytoplasmic inclusion body formation in autophagy-deficient mice. Cell 2007; 131: 1149–1163. 18083104 10.1016/j.cell.2007.10.035

[bib26] Liu WR, Tian MX, Yang LX, Lin YL, Jin L, Ding ZB et al. PKM2 promotes metastasis by recruiting myeloid-derived suppressor cells and indicates poor prognosis for hepatocellular carcinoma. Oncotarget 2015; 6: 846–861. 25514599 10.18632/oncotarget.2749 PMC4359260

[bib27] Peng XC, Gong FM, Zhao YW, Zhou LX, Xie YW, Liao HL et al. Comparative proteomic approach identifies PKM2 and cofilin-1 as potential diagnostic, prognostic and therapeutic targets for pulmonary adenocarcinoma. PLoS ONE 2011; 6: e27309. 22087286 10.1371/journal.pone.0027309 PMC3210781

[bib28] Yuan C, Li Z, Wang Y, Qi B, Zhang W, Ye J et al. Overexpression of metabolic markers PKM2 and LDH5 correlates with aggressive clinicopathological features and adverse patient prognosis in tongue cancer. Histopathology 2014; 65: 595–605. 24762230 10.1111/his.12441

[bib29] Yang W, Xia Y, Ji H, Zheng Y, Liang J, Huang W et al. Nuclear PKM2 regulates beta-catenin transactivation upon EGFR activation. Nature 2011; 480: 118–122. 22056988 10.1038/nature10598 PMC3235705

[bib30] Wong N, Ojo D, Yan J, Tang D. PKM2 contributes to cancer metabolism. Cancer Lett 2015; 356(2 Pt A): 184–191. 24508027 10.1016/j.canlet.2014.01.031

[bib31] Yamada K, Noguchi T. Nutrient and hormonal regulation of pyruvate kinase gene expression. Biochem J 1999; 337(Pt 1): 1–11. 9854017 PMC1219928

[bib32] Tang R, Yang C, Ma X, Wang Y, Luo D, Huang C et al. MiR-let-7a inhibits cell proliferation, migration, and invasion by down-regulating PKM2 in gastric cancer. Oncotarget 2016; 7: 5972–5984. 26745603 10.18632/oncotarget.6821 PMC4868734

[bib33] He Y, Wang Y, Liu H, Xu X, He S, Tang J et al. Pyruvate kinase isoform M2 (PKM2) participates in multiple myeloma cell proliferation, adhesion and chemoresistance. Leuk Res 2015; 39: 1428–1436. 26453405 10.1016/j.leukres.2015.09.019

[bib34] Luo W, Hu H, Chang R, Zhong J, Knabel M, O’Meally R et al. Pyruvate kinase M2 is a PHD3-stimulated coactivator for hypoxia-inducible factor 1. Cell 2011; 145: 732–744. 21620138 10.1016/j.cell.2011.03.054 PMC3130564

[bib35] Semenza GL. HIF-1: upstream and downstream of cancer metabolism. Curr Opin Genet Dev 2010; 20: 51–56. 19942427 10.1016/j.gde.2009.10.009 PMC2822127

[bib36] Peng X, Gong F, Chen Y, Jiang Y, Liu J, Yu M et al. Autophagy promotes paclitaxel resistance of cervical cancer cells: involvement of Warburg effect activated hypoxia-induced factor 1-alpha-mediated signaling. Cell Death Dis 2014; 5: e1367. 25118927 10.1038/cddis.2014.297 PMC4454295

[bib37] Warr MR, Binnewies M, Flach J, Reynaud D, Garg T, Malhotra R et al. FOXO3A directs a protective autophagy program in haematopoietic stem cells. Nature 2013; 494: 323–327. 23389440 10.1038/nature11895 PMC3579002

[bib38] Chiacchiera F, Simone C. Inhibition of p38alpha unveils an AMPK-FoxO3A axis linking autophagy to cancer-specific metabolism. Autophagy 2009; 5: 1030–1033. 19587525 10.4161/auto.5.7.9252

[bib39] Ho KK, Myatt SS, Lam EW. Many forks in the path: cycling with FoxO. Oncogene 2008; 27: 2300–2311. 18391972 10.1038/onc.2008.23

[bib40] Chiacchiera F, Simone C. The AMPK-FoxO3A axis as a target for cancer treatment. Cell Cycle 2010; 9: 1091–1096. 20190568 10.4161/cc.9.6.11035

[bib41] Wong HH, Jiang G, Gangeswaran R, Wang P, Wang J, Yuan M et al. Modification of the early gene enhancer-promoter improves the oncolytic potency of adenovirus 11. Mol Ther 2012; 20: 306–316. 22086234 10.1038/mt.2011.242 PMC3272483

[bib42] Liu TC, Hallden G, Wang Y, Brooks G, Francis J, Lemoine N et al. An E1B-19 kDa gene deletion mutant adenovirus demonstrates tumor necrosis factor-enhanced cancer selectivity and enhanced oncolytic potency. Mol Ther 2004; 9: 786–803. 15194046 10.1016/j.ymthe.2004.03.017

[bib43] Puig-Saus C, Gros A, Alemany R, Cascallo M. Adenovirus i-leader truncation bioselected against cancer-associated fibroblasts to overcome tumor stromal barriers. Mol Ther 2012; 20: 54–62. 21863000 10.1038/mt.2011.159 PMC3255593

[bib44] Tholey RM, Lal S, Jimbo M, Burkhart RA, Blanco FF, Cozzitorto JA et al. MUC1 promoter-driven DTA as a targeted therapeutic strategy against pancreatic cancer. Mol Cancer Res 2015; 13: 439–448. 25336517 10.1158/1541-7786.MCR-14-0199

[bib45] Goldenberg MM. New drugs/drug news. P T 2013; 38: 313–351.23946625

[bib46] Andtbacka RH, Kaufman HL, Collichio F, Amatruda T, Senzer N, Chesney J et al. Talimogene laherparepvec improves durable response rate in patients with advanced melanoma. J Clin Oncol 2015; 33: 2780–2788. 26014293 10.1200/JCO.2014.58.3377

[bib47] Puzanov I, Milhem MM, Minor D, Hamid O, Li A, Chen L et al. Talimogene Laherparepvec in Combination With Ipilimumab in Previously Untreated, Unresectable Stage IIIB-IV Melanoma. J Clin Oncol 2016; 34: 2619–2626. 27298410 10.1200/JCO.2016.67.1529 PMC7189507

[bib48] Kicielinski KP, Chiocca EA, Yu JS, Gill GM, Coffey M, Markert JM. Phase 1 clinical trial of intratumoral reovirus infusion for the treatment of recurrent malignant gliomas in adults. Mol Ther 2014; 22: 1056–1062. 24553100 10.1038/mt.2014.21 PMC4015229

[bib49] Ding M, Cao X, Xu HN, Fan JK, Huang HL, Yang DQ et al. Prostate cancer-specific and potent antitumor effect of a DD3-controlled oncolytic virus harboring the PTEN gene. PLoS One 2012; 7: e35153. 22509396 10.1371/journal.pone.0035153 PMC3324420

[bib50] Booth LA, Tavallai S, Hamed HA, Cruickshanks N, Dent P. The role of cell signalling in the crosstalk between autophagy and apoptosis. Cell Signal 2014; 26: 549–555. 24308968 10.1016/j.cellsig.2013.11.028 PMC4054685

[bib51] Djavaheri-Mergny M, Maiuri MC, Kroemer G. Cross talk between apoptosis and autophagy by caspase-mediated cleavage of Beclin 1. Oncogene 2010; 29: 1717–1719. 20101204 10.1038/onc.2009.519

[bib52] Zhu Y, Zhao L, Liu L, Gao P, Tian W, Wang X et al. Beclin 1 cleavage by caspase-3 inactivates autophagy and promotes apoptosis. Protein Cell 2010; 1: 468–477. 21203962 10.1007/s13238-010-0048-4 PMC4875131

[bib53] Wirawan E, Vande Walle L, Kersse K, Cornelis S, Claerhout S, Vanoverberghe I et al. Caspase-mediated cleavage of Beclin-1 inactivates Beclin-1-induced autophagy and enhances apoptosis by promoting the release of proapoptotic factors from mitochondria. Cell Death Dis 2010; 1: e18. 21364619 10.1038/cddis.2009.16 PMC3032505

[bib54] Eisenberg-Lerner A, Bialik S, Simon HU, Kimchi A. Life and death partners: apoptosis, autophagy and the cross-talk between them. Cell Death Differ 2009; 16: 966–975. 19325568 10.1038/cdd.2009.33

