# Supplementary material for: RGD-modified oncolytic adenovirus-harboring shPKM2 exhibits a potent cytotoxic effect in pancreatic cancer via autophagy inhibition and apoptosis promotion
Source: Cell Death Dis. 2017 Jun 1;8(6):e2835–. doi: 10.1038/cddis.2017.230 (PMC5520890; doi:10.1038/cddis.2017.230)
Supplement: Supplementary Figure legends [file cddis2017230x1.docx]

**Supplementary Figure legends**

**Supplementary Figure S1.** (a, b) qRT-PCR (a) and western blot (b) showed unaltered levels of PKM1 in PANC-1 and BxPC-3 cells stably expressing shPKM2. (c) The western blot results indicated reduced expression of cyclin D1 in PANC-1 and BxPC-3 cells stably expressing shPKM2. (d, e) PKM2 knockdown inhibited cell growth as measured by cell counting. (f) Representative images of the histological analysis of tumor and liver sections of xenograft tumors comprising PANC-1 or PANC-1 cells stably expressing shPKM2-1. Scale bar, 200 μm (upper); 100 μm (lower). (g) Representative images of metastatic liver nodules (arrow) in mice subcutaneously injected with either control or shPKM2-1-expressing PANC-1 control cells. (h) Serum AST and ALT levels were measured after mice were euthanized. AST, aspartate aminotransferase; ALT, alanine aminotransferase. All the experiments were repeated three times, and the bars represent the mean ± S.D. (n = 3 in a; n = 4 in h; n = 6 in d, e). *p < 0.05, **p < 0.01. NS, no significance.

**Supplementary Figure S2.** (a) Representative images of LC3 puncta in PANC-1 and BxPC-3 cells stably expressing shPKM2. Scale bars, 10 μm. (b, c) Statistical data of ten repeats of (a). (d) Representative electron microscopy images (scale bar, 500 nm) of PANC-1 and BxPC-3 cells stably expressing shPKM2. (e-g) qRT-PCR (e, f) and western blot (g) showed a reduction in HIF-1α and FoxO3a expression in PANC-1 and BxPC-3 cells stably expressing shPKM2. (h) PKM2 knockdown did not induce apoptosis in PANC-1 and BxPC-3 cells; however, apoptosis was detected in cells with stable PKM2 knockdown as determined using annexin V/PI staining. The qRT-PCR data in (e, f) were normalized to 18S RNA levels. The bars represent the mean ± S.D. (n = 10 in b, c; n = 3 in e, f). *p < 0.05, **p < 0.01.

**Supplementary** **Figure S3.** (a) Flow cytometry analysis of CAR expression in pancreatic cancer cells and the hepatocellular carcinoma cell line QGY-7701. (b) Quantification of the percentage of CAR-positive cells in (a). (c) Schematic of oncolytic adenovirus construction. Arrows indicate the primers used for identification of wild-type adenovirus contamination. (d) Sequence of oncolytic adenoviruses for verification of RGD insertion. (e) The E1B deletion was verified by PCR using the primers listed in Figure 4a. The arrow indicates the wild-type E1B fragment. (f, g) The percentage of EGFP-positive cells was significantly increased in PANC-1 cells treated with O^Ad^.R.EGFP for 2 days than in cells treated with O^Ad^.EGFP. The number of EGFP-positive cells was detected by fluorescence-activated cell sorting analysis. (h) Tumors were excised and photographed 35 days after injection. (i) Serum AST and ALT levels were measured after the mice were euthanized. AST, aspartate aminotransferase; ALT, alanine aminotransferase. The bars represent the mean ± S.D. (n = 3 in b, g; n = 6 in i).

**Supplementary Figure S4.** (a) Representative images of LC3 puncta in PANC-1 cells infected with the indicated oncolytic adenoviruses. Scale bars, 10 μm. (b) Statistical data of ten repeats of (a). The bars represent the mean ± S.D. (n = 10 in b). *p < 0.05, **p < 0.01. (c) Representative electron microscopy images (scale bar, 500 nm) of PANC-1 cells infected with the indicated oncolytic adenoviruses. (d) Apoptosis was evaluated in PANC-1 cells infected with oncolytic adenovirus using annexin V/PI staining. Representative images of the flow cytometry analysis are shown. (e) Apoptosis was evaluated using annexin V/PI staining in cells with stable PKM2 knockdown that were treated with O^Ad^.R at an MOI of 10 for 2 days. Representative images of the flow cytometry analysis are shown.

**Supplementary Figure S5.** (a) PKM1 expression was analysed by IHC in the pancreatic cancer tissue (CT, n = 198) and adjacent noncancerous tissue specimens (ANCT, n = 84). (b) Representative IHC images. The scale bars represent 50 μm. (c) Kaplan–Meier analysis to plot the correlation between PKM1 expression and overall survival of 60 pancreatic cancer patients. (d, e) PKM1 expression of the pancreatic cancer cell lines AsPC-1, PANC-1, BxPC-3, and CFPAC-1 and the normal pancreatic duct cell line hTERT-HPNE was measured using qRT-PCR (d) and western blotting (e). The qRT-PCR data in (d) were normalized to 18S RNA levels. All the experiments were repeated three times, and the bars represent the mean ± S.D. (n = 3), NS, no significance.
