# Supplementary material for: RGD-modified oncolytic adenovirus-harboring shPKM2 exhibits a potent cytotoxic effect in pancreatic cancer via autophagy inhibition and apoptosis promotion
Source: Cell Death Dis. 2017 Jun 1;8(6):e2835–. doi: 10.1038/cddis.2017.230 (PMC5520890; doi:10.1038/cddis.2017.230)
Supplement: Supplementary Table S1 [file cddis2017230x7.docx]

**Additional file 5: Table S1. Sequences of oligonucleotide primers**

| **Primers** | **Sequences** |
| --- | --- |
|  |  |
| WT-forward | 5’- GCAACCTTTGGACTTGAGCTGT-3’ |
| WT-reverse | 5’-TCTCATCGTACCTCAGCACCTTC-3’ |
|  |  |
| PKM2-forward | 5’-ATTATTTGAGGAACTCCGCCGCCT-3’ |
| PKM2-reverse | 5’- ATTCCGGGTCACAGCAATGATGG-3’ |
|  |  |
| RGD-forward | 5’- GCTTGAGGTTAACCTAAGCACT-3’ |
| RGD-reverse | 5’- AAATGACTTGAAATTTTCTGCAAT-3’ |
|  |  |
| 18S-forward | 5’- AACTTTCGATGGTAGTCGCCG-3’ |
| 18S-reverse  HIF-1 α -forward  HIF-1 α -reverse  FoxO3a -forward  FoxO3a -reverse  PKM1-forward  PKM2-reverse | 5’- CCTTGGATGTGGTAGCCGTTT-3’  5’-GGACAGAGTAGTTCCAGAGGCAGTTC-3’  5’-GGTGTGCATTTCCACATCAAACAT-3’  5’-CATCTCCACCTTCTACCC-3’  5’-CTCTTTCCTGCTCTGTCTG-3’  5’-CGAGCCTCAAGTCACTCCAC-3’  5’-GTGAGCAGACCTGCCAGACT-3’ |
